# Scalable Model for Delivery of Inpatient Palliative Care During a Pandemic

**DOI:** 10.1177/10499091211005701

**Published:** 2021-04-07

**Authors:** Ebru Kaya, Warren Lewin, David Frost, Breffni Hannon, Camilla Zimmermann

**Affiliations:** 1 Department of Supportive Care, 7989 University Health Network, Toronto, ON, Canada; 2 Division of Palliative Medicine, Department of Medicine, 7989 University Health Network, Toronto, ON, Canada; 3 Division of Palliative Care, Department of Family and Community Medicine, 7989 University Health Network, Toronto, ON, Canada; 4 Division of General Internal Medicine, Department of Medicine, 7989 University Health Network, Toronto, ON, Canada; 5 Division of General Internal Medicine, Department of Medicine, 7989 University of Toronto, ON, Canada

**Keywords:** pandemic, COVID-19, novel coronavirus, inpatient model, palliative care, scalable

## Abstract

**Background:**

During the COVID-19 pandemic, hospitals worldwide have reported large volumes of patients with refractory symptoms and a large number of deaths attributable to COVID-19. This has led to an increase in the demand for palliative care beyond what can be provided by most existing programs. We developed a scalable model to enable continued provision of high-quality palliative care during a pandemic for hospitals without a palliative care unit or existing dedicated palliative care beds.

**Methods:**

A COVID-19 consultation service working group (CWG) was convened with stakeholders from palliative care, emergency medicine, critical care, and general internal medicine. The CWG connected with local palliative care teams to ensure a coordinated response, and developed a model to ensure high-quality palliative care provision.

**Results:**

Our 3-step scalable model included: (1) consultant model enhanced by virtual care; (2) embedded model; and (3) cohorted end-of-life unit for COVID-19 positive patients. This approach was enabled through tools and resources to ensure specialist palliative care capacity and rapid upskilling of all clinicians to deliver basic palliative care. Enabling tools and resources included a triage tool for in-person versus virtual care, new medication order sets and guidelines to facilitate prescribing for common symptoms, and lead advance care planning and goals of care discussions. A redeployment plan of generalist physicians and psychiatrists was created to ensure seamless provision of serious illness care.

**Conclusion:**

This 3-step, scalable approach enables rapid upscaling of palliative care in collaboration with generalist physicians, and may be adapted for future pandemics or natural disasters.

## Introduction

COVID-19, caused by the novel coronavirus SARS-CoV2, was first described in December 2019 and was declared a global pandemic by the World Health Organization in March 2020.^1^ Patients hospitalized with COVID-19 may have severe and potentially refractory symptoms, including shortness of breath and delirium^2^ and may rapidly deteriorate requiring expert end-of-life care. As well, goals of care (GOC) conversations are needed for these patients in the hospital setting to direct treatment decisions and have been a key reason for referral to palliative care teams during the pandemic.^3^ As palliative care shifts from only being offered at the end of life to earlier in the disease trajectory, coupled with an older population with multiple chronic illnesses and the existing shortage of specialist palliative care providers worldwide,^4,5^ it is unlikely that specialist palliative care teams will be able to provide direct care for all hospitalized patients with COVID-19 who have palliative care needs, particularly in the case of a surge of admitted patients and/or reduced staffing due to clinician illness or quarantine.

In planning our local response to the COVID-19 pandemic, we developed a 3-stage scalable approach aimed at providing the best possible palliative care, depending on the number of patients requiring these services. Here we describe this approach, which may be used to rapidly upscale palliative care during a pandemic for palliative care teams practicing in the acute-care setting.

## Methods

### Setting

The Toronto General Hospital and the Toronto Western Hospital are large academic tertiary care hospitals, which are part of the University Health Network (UHN) in Toronto, Canada. Patients with COVID-19 are cared for on dedicated units staffed by general internal medicine interprofessional teams at the acute care hospitals. At both hospitals, around-the-clock consultative palliative care is provided by physicians and an advanced practice nurse to inpatients, outpatients, and to patients presenting to the emergency departments; however, neither hospital has an inpatient palliative care unit or dedicated palliative care beds. Inpatients have diagnoses ranging from advanced cancer to chronic non-cancer diseases such as heart failure, end stage lung, liver and kidney disease. Our palliative care consultation services assist with the assessment and treatment of symptoms, advance care planning (ACP), GOC discussions, and disposition planning.

### Convening a Group

A COVID-19 consultation service working group (CWG) was convened at the early stages of the pandemic, comprising key stakeholders including palliative care, emergency medicine, critical care, general internal medicine, and COVID-19 clinical and administrative hospital clinicians leading the health system’s pandemic response. The CWG was tasked with reviewing and synthesizing known data from the biomedical literature; connecting with local palliative care teams in the area to ensure a coordinated response; and providing clinical practice recommendations to the health system based on prior experiences and existing tools. The committee worked closely with and reported to the department’s COVID-19 pandemic committee, which had representation at the senior executive leadership level to ensure that the proposed response aligned with the institution’s coordinated pandemic response.

### Establishing a Common Goal

The overarching goal was to ensure seamless and ongoing assessment and management of palliative care needs from the emergency department and throughout hospitalization until discharge or death for a surge of patients with COVID-19 and their families receiving care in our health system.

## Results

Based on stakeholder discussions, 2 tasks were prioritized: (1) ensuring direct specialist palliative care delivery remained available for patients and families with specialist needs and who were dying from COVID-19, and (2) rapid upskilling for non-palliative care clinicians to manage common symptoms related to COVID-19, and to lead ACP and GOC discussions to ensure care remained aligned to patient and family values and preferences.

**Figure 1.**
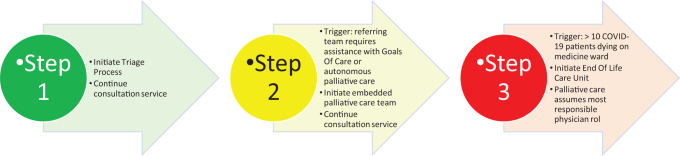
Scalable model for palliative care during a pandemic.

**Figure 2.**
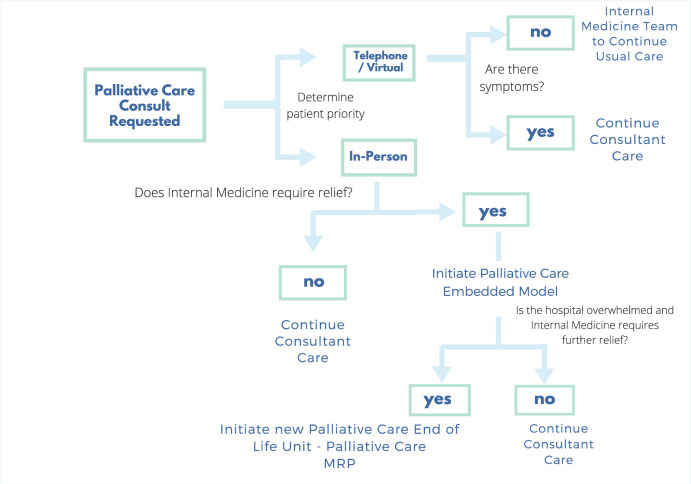
Algorithm for palliative care team workflow during COVID-19.

To address priority 1, we developed a 3-stage, stepwise approach to increase the ability of our consultation services to meet the palliative needs of admitted patients depending on volumes of patients admitted during the pandemic and based on available non-specialists to continue providing care (Figures 1 and 2).

### Stage 1—Consultant Model With Virtual Option

The health system directed in-person care to be based on need, to minimize the risk for patients, families, and staff alike to transmit or acquire COVID-19. As such, in Stage 1, referring providers continued to make requests for inpatient palliative care consultations, but were notified of the possibility for patients to receive virtual care by telehealth instead of usual in-person care, depending on patient/family need as well as staffing availability. We developed triage criteria (Table 1) to assist with prioritizing patients requiring in-person versus virtual care, based on severity of palliative care needs. For inpatient virtual consultations, the institution’s wireless internet service was provided free of charge and iPads were purchased by the institution for use at the bedside for inpatients without a personal smart device. In addition, we provided 24/7 telephone coaching to empower clinical services to provide primary palliative care. While patient volumes remained manageable, the palliative care team focused on building basic palliative care capacity for our non-specialist colleagues (see next section).

**Table 1.** Triage Criteria for In-Person Versus Virtual Inpatient Consultation.

In person consultation	Virtual consultation
Severe symptoms refractory to initial management by referring service.	Symptomatic patients not meeting “In Person” criteria.
Patients on or requiring an infusion pump for refractory pain, shortness of breath, agitation.	Patients and families experiencing psychosocial distress relating to transition to palliative care philosophy.
Patients experiencing intolerance or adverse effects from opioid therapy.	Referring team requires support and coaching with respect to goals of care conversations (i.e., identifying disease understanding, hopes, expectations, values, acceptable trade-offs, code status, desired location of death, etc.)
Patients with refractory symptoms who are actively dying.	
Goals of care discussion requiring specialist palliative care input regarding code status or acute treatment	Most responsible physician requires support and coaching for palliative planning such as palliative nursing home care referral, referral to a palliative care physician in the community, or applications to palliative care units or hospices.
Decision potentially impacting location of care for a patient at risk for decompensation and possible transfer to intensive care unit.	

### Stage 2—Embedded Model

As the surge worsened, patients who were admitted for management of their COVID-19 received care on dedicated units staffed by an interprofessional team. Stage 2 was triggered when the most responsible physician managing a COVID unit requested greater assistance with GOC discussions or increased autonomy of the palliative care team for symptom management due to a surge of dying patients on the COVID wards. Members from the palliative care team were embedded into these hospital teams (e.g., emergency department, intensive care unit, internal medicine teams) and rounded daily with them to assist with the identification of unmet palliative care needs including patients that could benefit from GOC discussions and identification and documentation of substitute decision makers. The palliative care team ensured that substitute decision makers and discussions with patients and family were documented clearly in the patient’s chart to assist with treatment decisions. An embedded model of care was selected to maximize daily communication between interdisciplinary clinicians, have the same palliative care team member on the ward only once daily to minimize team exposure risk, and allow the embedded team member to build rapport and trust with the MRP teams.

### Stage 3—Cohorted End-of-life Care Unit

Stage 3 was designed to be triggered when the surge was worsening to the point where clinicians were concerned that they could not provide safe patient care or when most the patients admitted to a COVID ward were expected to die in the hospital during their admission. For patients who met the criteria for admission to a typical palliative care unit in Toronto for end-of-life care,^6^ palliative care physicians provided coverage as their MRP, while after-hours telephone coverage was provided by the internal medicine team. The palliative care team continued to attend daily rounds with the internal medicine COVID teams to help them to identify patients with care needs that could be managed more autonomously by palliative care clinicians. This increased the availability for non-palliative care physicians to provide additional surge-related care while creating a space for both teams to discuss challenges and support each other. Cohorting the patients allowed for preservation of PPE, reduced clinician foot traffic, and allowed specialist palliative care nurses to provide education and support on internal medicine wards to better manage dying patients’ needs.

Stage 3 also triggered the redeployment of family physicians, geriatric specialists and psychiatrists to join the palliative care team by leading ACP or GOC conversations in the emergency department and hospital wards for patients either diagnosed with COVID, suspected of having COVID or at risk to die during their hospitalization as determined by the admitting or emergency department clinician. This redeployment to palliative care was part of the larger redeployment plan for our institution, designed to rapidly provide education and skill-building to enable the delivery of high-quality serious illness conversations, and was enabled by the creation of a repository of both existing and newly developed education tools and standardized templates described below.

### Rapid Capacity Building for Primary Palliative Care

Despite preparing to assist with symptom control during a surge, the main palliative intervention addressed a pre-existing knowledge and skill gap: how to lead a values-based conversation to ensure care was aligned with goals. With COVID-19, it was necessary to rapidly build capacity for primary palliative care, both for non-palliative care clinicians providing primary care, and for clinicians redeployed to help with providing consultant palliative care on palliative care teams. Below we describe tools and resources that were developed to enable this effort.

### Education Initiatives

Webinars and slide decks were created with local education experts via an online platform accessible to clinicians free of charge.^7^ Topics were separated into categories for ease of use and included: i) A-Z of palliative care with a primer as well as an introduction to the general philosophy, benefits and myths related to palliative care; ii) sections on caring for dying patients with practical tips, such as when to refer to specialist palliative care and how to complete a death certificate; and iii) communication skills with scripted language based on pre-existing evidence-based tools but modified for use during the pandemic.

### Guidelines and Resources

Symptom guidelines were developed with specific consideration given to the management of respiratory symptoms and the care of patients at the end of life, including palliative sedation. These resources ranged from comprehensive guidelines to 1-page pocket guides with clear instructions for appropriate medication choices, doses and associated frequency and route of administration. Corresponding order sets and tools for documenting substitute decision makers in various languages, and goals of care discussions were developed for use at all sites, in order to streamline the process for documenting discussions and treatment plans and entering orders for rapid symptom management. They included consideration of potential drug shortages, based on local information and emerging data from other countries.^8^

### Staffing Considerations

To ensure adequate staffing across inpatient and outpatient palliative care services, we rescheduled non-urgent outpatient appointments and arranged for outpatient palliative care to be provided virtually, where possible. If staff on the inpatient service became ill or exposed to COVID-19, requiring home quarantine, we had the capability to mobilize our outpatient team to cover inpatient staff shortages. Residents and fellows were aligned with faculty and back-up schedules were created for on-call coverage.

We also created “geographically distinct” teams within each site to minimize possible viral transmission to and between team members. Staff were assigned to see patients either with or without COVID-19, but not both. Those seeing patients without COVID-19 were prioritized to provide care on the COVID-negative inpatient palliative care unit, given its vulnerable patient population. “Geographically distinct” coverage also minimized time spent donning and doffing PPE.

Finally, we collaborated with interdepartmental colleagues to develop a redeployment plan that included adding family medicine, geriatric medicine specialists and psychiatrists to our team, if needed. These clinicians were prioritized for palliative care redeployment, as their general medical and communication skills were felt to be most closely aligned to the field of palliative care and they could be trained and coached quickly, to conduct ACP and GOC conversations directly in the emergency department.

## Discussion

We developed an inpatient palliative care response to the COVID-19 pandemic that can be used by teams without an inpatient palliative care unit, or where the number of patients with COVID-19 exceeds the capacity of the existing palliative care unit. We utilized data from other countries’ experiences with COVID-19^9^ as well as data relating to previous pandemics^10^ to inform our response. While palliative care management approaches were mostly unchanged, new structures and strategies were developed to ensure patients’ needs were met.

Other models of palliative care have also been successfully implemented during the COVID-19 pandemic. Many of these share common elements with our model, including the development and dissemination of symptom management and communication toolkits for non-specialist providers^3,11,12^; triaging consultation referrals based on need^3,11^; offering virtual consultations where feasible to preserve PPE and minimize risk of exposure^3,11-13^; embedding palliative care clinicians within teams in the emergency department, intensive care unit and acute care services^11^; and splitting teams within sites to maintain a core group of healthy clinicians in the event of exposure.^3^ A different model focused more on the development and widespread dissemination of resources across acute and community settings^12^; this model may be more relevant to services covering a large or diverse geographical area, and where the regular movement of clinicians between and within settings may have been curtailed by the risk of exposure or lack of PPE.

While the Delphi technique or others are commonly used in the development of new models or ideas, they are typically labor-intensive. Completing a comprehensive literature review, recruiting a panel of appropriate stakeholders, collecting pre- and post-panel data, assimilating this data into recommendations and revising these based on further stakeholder feedback, before finally developing a consensus tool or document can take many months to complete.^14,15^ For our model, the rapid declaration of the pandemic and need to develop immediate responses and strategies meant that there was insufficient time to convene a traditional Delphi panel; instead, we assimilated a convenience sample of clinical and administrative leaders from within our organization to direct a response to best meet the needs of our patients as well as our clinical teams.

The intent of this paper was to describe our 3-step model of care delivery during the COVID-19 pandemic. This model was developed to ensure timely access to specialized palliative care for patients with COVID-19 who need it most, while simultaneously ensuring that all patients with and without COVID-19 have ongoing access to impeccable symptom management and comprehensive goals of care discussions. A further paper will describe in detail the results of this implementation, using administrative data. This model may be adapted for future pandemic surges or natural disasters for other centers internationally.
